# How Do Test Takers Interact With Simulation-Based Tasks? A Response-Time Perspective

**DOI:** 10.3389/fpsyg.2019.00906

**Published:** 2019-04-24

**Authors:** Yi-Hsuan Lee, Jiangang Hao, Kaiwen Man, Lu Ou

**Affiliations:** ^1^Educational Testing Service, Princeton, NJ, United States; ^2^Department of Human Development and Quantitative Methodology, Measurement, Statistics and Evaluation Program, University of Maryland at College Park, College Park, MD, United States; ^3^ACT Inc., Iowa City, IA, United States

**Keywords:** trialogue, response time, hierarchical modeling framework, cluster analysis, motivation, rapid-guessing behavior

## Abstract

Many traditional educational assessments use multiple-choice items and constructed-response items to measure fundamental skills. Virtual performance assessments, such as game- or simulation-based assessments, are designed recently in the field of educational measurement to measure more integrated skills through the test takers’ interactive behaviors within an assessment in a virtual environment. This paper presents a systematic timing study based on data collected from a simulation-based task designed recently at Educational Testing Service. The study is intended to understand the response times in complex simulation-based tasks so as to shed light on possible ways of leveraging response time information in designing, assembling, and scoring of simulation-based tasks. To achieve this objective, a series of five analyses were conducted to first understand the statistical properties of the timing data, and then investigate the relationship between the timing patterns and the test takers’ performance on the items/task, demographics, motivation level, personality, and test-taking behaviors through use of different statistical approaches. We found that the five analyses complemented each other and revealed different useful timing aspects of this test-taker sample’s behavioral features in the simulation-based task. The findings were also compared with notable existing results in the literature related to timing data.

## Introduction

Many traditional educational assessments use multiple-choice (MC) items and constructed-response (CR) items to measure fundamental skills, such as verbal and quantitative skills. The MC and CR items in the same form are assembled to measure the same construct but usually are not attached to a common scenario throughout the test. There is an increasing interest in the field of educational measurement in developing new capabilities for new task formats and assessment types to measure more integrated skills, such as problem-solving and critical thinking, which may not be directly assessed by those traditional educational assessments. Virtual performance assessments (VPAs), such as game- or simulation-based assessments, are often used to serve the purpose (Baker and Clarke-Midura, 2013; Mislevy et al., 2014). In a VPA, a test taker’s proficiency is assessed based on his/her interactions with the virtual environment. As such, good understanding of how the test taker interacts with the virtual environment is essential for developing psychometrically sound scoring rules for VPAs,and for designing and assembling VPAs to support the intended scoring rules. In this paper, we aim at better understanding the test taker’s interactions with the virtual environment from the perspective of their response time (RT) to the items in a VPA.

There is rich literature on RT research concerning the design, assembly, and scoring aspects of traditional MC tests that are digitally based (for review papers, see, e.g., Schnipke and Scrams, 2002; Lee and Chen, 2011; Kyllonen and Zu, 2016; De Boeck and Jeon, 2019). These literature also suggests that RTs contain rich information about test takers’ response processes, test-taking behaviors and strategies, and motivation. One reason is that test takers’ timing behaviors reflect person-task interactions. When the major assessment outcomes to be scored are the final responses to items, test takers may adjust their timing behaviors or strategies to cope with the test conditions in order to optimize their test performance. The adjustment in behavior or strategy may occur before people take a test (during practice exams) or during a live test (Lee and Haberman, 2016). Thus, compared to item responses, their timing behaviors tend to be more sensitive to test context and content, test/item type, and test conditions. RTs have been used as ancillary information for improving precision of parameter estimation and validity of measurement beyond what is available based on item responses: For example, for tests that are intended to measure both speed and accuracy, RTs may be used to derive scores together with item responses (Maris and van der Maas, 2012; van Rijn and Ali, 2018). To have better control on test speededness, RTs may be utilized for assembling test forms in non-adaptive testing and selecting items in adaptive testing (e.g., van der Linden et al., 1999; Choe et al., 2018). In addition, RTs have been used in test security analyses and examination of general test-taking behaviors (e.g., solution behavior vs. rapid-guessing behavior, due to test speededness or low motivation).

To our knowledge, test takers’ timing behaviors in VPAs have been less explored psychometrically, possibly due to limited access to large-scale empirical data from VPAs. Educational Testing Service (ETS) researchers have conducted a timing study of simulation-based tasks in the context of the National Assessment of Educational Progress (NAEP; Jia and Lee, 2018). The study focused on two simulation-based tasks, each with four items given with a time limit to around 2,000 students; the tasks assessed technology and engineering literacy of grade eight students in the United States. This study had three primary findings. First, the items that asked the students to conduct simulations or experiments (referred to as *simulation items* henceforth) required much more time to complete than the rest of the items did, but the simulation items did not appear to be especially difficult. Second, rapid-guessing behavior was not an issue for these simulation-based tasks, although the assessment was considered low-stakes to the students. Third, the correlation between the observed task time and performance was positive but almost negligible. Note that each of the two NAEP simulation-based tasks was used as part of a test form for assessing technology and engineering literacy and the scores were not reported at the task level. While RTs have also been examined in other fields, the focuses tend to be different from those in educational measurement—for example, to study varying student interactions in computer-supported collaborative learning (e.g., Jeong, 2004) or to assess learning in intelligent tutoring systems (e.g., Beck et al., 2000).

The simulation-based tasks considered in Jia and Lee (2018) were relatively short and simple. In this current work, we furthered the effort on RT analysis to study a more complex simulation-based task that has a complete storyline about how a test taker investigates volcano eruption in a virtual geology lab. This simulation-based task was developed as part of an effort to assessing collaborative problem-solving (CPS) skills in science, the ETS Collaborative Science Assessment Prototype (ECSAP; Hao et al., 2015, 2017; Liu et al., 2015). In ECSAP, there are two parallel simulation-based tasks. One is intended for individual test takers to respond, referred to as the single-user version. The other is for dyadic teams to respond collaboratively, referred to as the collaborative version. Both the individual and collaborative versions of the simulation-based tasks were modified from an earlier simulation-based task about volcano science designed to assess students’ science inquiry skill (Zapata-Rivera et al., 2014). In the single-user version, each participant responded to 11 items without any time limit, and their item responses and item RTs were captured. In the collaborative version, two human participants collaborated through a chat box to interact with two virtual agents to complete the same task. In the previous research, the foci were primarily on the collaborative version of the simulation-based task to explore CPS skills and collaboration engagement through the online chats (e.g., content, frequency, and chat time) between team members and their item responses (see the CPS references above, and Halpin et al., 2017), while the single-user version was simply used as a control. No systematic timing analysis has been carried out using data collected from either version of the tasks.

In this paper, we present a systematic study on the RTs collected from the single-user version of the simulation-based task. Our goal is to understand the RTs in complex simulation-based tasks so as to shed light on possible ways of leveraging RT information in designing, assembling, and scoring of simulation-based tasks. To achieve the objective, a series of five analyses were conducted to first understand the statistical properties of the timing data, and then investigate the relationship between the timing patterns and the test takers’ performance on the items/task, demographics, motivation level, personality, and test-taking behaviors through use of different statistical approaches. As will be shown, the five analyses complement each other and reveal different timing aspects of this test-taker sample’s behavioral features in the simulation-based task we studied. The behavioral features observed in this simulation-based task may be quite different from those in traditional educational assessments, and the comparisons will benefit RT researchers as well as researchers who are interested in the same or similar datasets.

It is worth noting that the study concentrates on timing and response data in the simulation-based task, although in general, a simulation-based task may have many assessment metrics beyond RTs and responses that are worth exploring. Also, this study is not intended to evaluate the potential of simulation-based tasks or VPAs beyond timing and response data for use in the field of educational measurement. For more general discussion about VPAs, please see, for example, Baker and Clarke-Midura (2013) and Mislevy et al. (2014). The rest of the paper is organized as follows. The next section provides information about the simulation-based task and the data under study. The series of five analyses are then described in detail regarding the methods and results. The Discussion section concludes the findings, addresses the implications of the results for the design, assembly, and scoring of simulation-based tasks, and discusses possible directions for future research.

## Data

As mentioned earlier, this study is based on a secondary analysis of the existing data on the simulation-based task about volcano science published in Hao et al. (2015, 2017) and Liu et al. (2015). The simulation-based task (referred to as *the task* henceforth) was designed to measure science inquiry skills on volcano science and delivered to 463 test takers on Amazon Mechanical Turk. Each test taker interacted with two virtual agents to complete 11 items embedded in a common scenario. The task began with an introduction to scientific information about volcano eruptions, followed by seven selected-response items on knowledge assessment (Items 1–7), and then four CR items on a simulation (Items 8–11). Among the four items about the simulation, the test takers were supposed to conduct a simulation on Item 8, in which they had to decide on the number of seismometers they wanted to use to monitor the volcano and then placed them in different regions around the volcano to collect data; they were then asked to explain why they chose that number of seismometers and the time duration they wanted to collect the data on Items 9 to 11. Table 1 presents the type and format of the 11 items, with some details (e.g., number of options per MC item and what actions were required per CR item) that will be used in discussing the analysis results. In this study, all items were scored dichotomously as 0 (incorrect) or 1 (correct). For Item 8, the score was based on the correctness and completeness of the simulation. As will be shown, such items may not be difficult but are typically time-consuming. It is noteworthy that, as compared to traditional educational tests, the level of task complexity—in terms of multiple item types and formats, and the actions required to achieve a correct answer to the embedded items—is unusual. Thus, some findings in this study are likely unique to simulation-based tasks and not necessarily generalizable to traditional educational tests with MC and/or CR items.

**Table 1:** Information about the 11 items in the task.

Item	Type	Format	Note	Chance-level proportion correct
1	MC	Single selection	1 out of 4 options	1/4
2	MC	Single selection	1 out of 4 options	1/4
3	MC	Multiple selection	2 out of 3 options	1/3
4	MC	Order	order 5 options	1/120
5	MC	Single selection	1 out of 4 options	1/4
6	MC	Single selection	1 out of 4 options	1/4
7	MC	Single selection	1 out of 4 options	1/4
8	CR		Simulation item	0
9	CR		Explain the design	0
10	CR		Explain the design	0
11	CR		Explain the design	0

*The chance-level proportion correct refers to the expected probability of answering the item correctly by guessing. This information is used in Analysis 5.*

The test takers could only take the items in the delivery order and were not permitted to revisit earlier items in the task. There was no time limit imposed on the task and everyone completed the task, so the data involved no missing item responses and RTs. For each test taker, the overall task time comprised two portions—one portion involving the time spent listening to scientific information about the common scenario, and the other portion involving the time spent working on the embedded items. The former portion was a fixed amount of time paced by the system, and was ignored in the rest of the study. The latter portion consisted of the item RTs under evaluation. In this study, we chose to consider the item-level RTs as the starting point to navigate the person-task interactions in the task, together with item-level responses. This choice facilitated the comparison of findings across items within the simulation-based task, and between the simulation-based task and the traditional educational tests examined in the RT literature. In this paper, for each individual, the task score refers to the sum of the responses to the 11 items, and the task time refers to the sum of the 11 item RTs.

In addition to the task, the test takers also responded to a standalone test for general science knowledge (with 37 single-selection MC items, referred to as *the MC test* henceforth), a demographic survey (including questions about their motivation level when completing the task), and a 10-item personality survey (Gosling et al., 2003). For more details about these different task/test/surveys, see Hao et al. (2017). The scores on the MC test and the responses to the survey questions were available for 445 of the 463 test takers, and this additional information was used as person covariates in the study. Thus, data from the 445 test takers were used in all analyses. Below is some information about the composition of the test-taker sample under study:

(a) About 63.6% of them were male.
(b) Their age ranged from 18 to 51, with a median of 24.
(c) They could be classified into four major ethnic groups—White (75.5%), Asian (12.8%), Black (6.1%), and others (5.6%).
(d) Regarding their career plan after college^1^—about 70.8% planned to work or worked full time, about 22.3% planned to attend or attended graduate school, and the rest had other plans.
(e) On the three motivation questions—did you find the task engaging? Did you find the task interesting? Did you learn something new from the task?—the fractions of the test takers answering 1, 2, or 3 (from agreeing most to least) was about 60%, 35%, and 5%, respectively.

It is worth mentioning that the test takers on Amazon Mechanical Turk were recruited to complete the task, the MC test, and the surveys. The MC test had several items designed to monitor if the test takers paid enough attention to the test and that might affect the payment. One example item was as follows: Which of the following cannot be found on earth? (a) Ocean; (b) moon (key); (c) dessert; and (d) woods. Those items were so easy that any test taker in the sample who considered them were able to answer correctly. All of the test takers included in this study answered the attention-track items correctly. Thus, it is expected that the test takers would be motivated in completing the task to some extent, although they experienced no consequences for their performance on the task and the MC test.

## Analyses and Results

In this section, we present five analyses that were intended to investigate the following aspects of the task times and item RTs collected from the task:

(1) Statistical properties of the task times and item RTs.
(2) How did the task times relate to the test takers’ performance on the task/MC test, demographics, motivation level, and personality?
(3) How did the item RTs and responses relate to each other?
(4) Did the test takers show different timing patterns across items? Did they inform differences in strategies/time allocation on the task?
(5) Did the test takers show rapid-guessing behavior on this task? Was there a clear motivation issue in this dataset?

## Analysis 1: Statistical Properties of the Timing Data

Because the task was given without time limits, the first question to answer was how the task times and item RTs varied for different test takers. Descriptive statistics of task scores and item responses were evaluated to complement the timing analysis at different levels. In addition, how the timing variables were distributed was of interest, as later analyses involved modeling of task times and RTs.

### Methods

Basic summary statistics were computed for task times and item RTs. Boxplots were made to show possible differences in the RT distributions for the 11 items. Preliminary results suggested that the histogram of task times and the histograms of item RTs had unimodal, right-skewed shapes. Thus, the distribution of task times and the distributions of item RTs were examined via QQ-plots and the Kolmogorov-Smirnov test, with respect to three theoretical models with these properties—lognormal model, gamma model, and Weibull model. These are three popular parametric models in time-to-event studies in survival analysis (Kalbfleisch and Prentice, 2002). The Kolmogorov-Smirnov test is a non-parametric test of the equality of continuous probability distributions that can be used to compare the empirical distribution function of a sample with a reference (theoretical) probability distribution. The type I error rate was set at 0.05 for evaluating the Kolmogorov-Smirnov test results.

### Results

Regarding the task-level data, the task times were typically short, ranging from 1.5 to 18.3 min. The first quartile, the median, and the third quartile of the task times were equal to 3.3, 4.2, and 5.3, respectively. The task scores ranged from 0 to 11, with the first quartile, the median, and the third quartile of the task scores equal to 6, 8, and 9, respectively. Overall, the test takers had decent performance on the items without spending much time. These test takers also performed well on the MC test, with the middle 50% of test takers scoring between 25 and 32 on a 0–37 scale.

**FIGURE 1:**
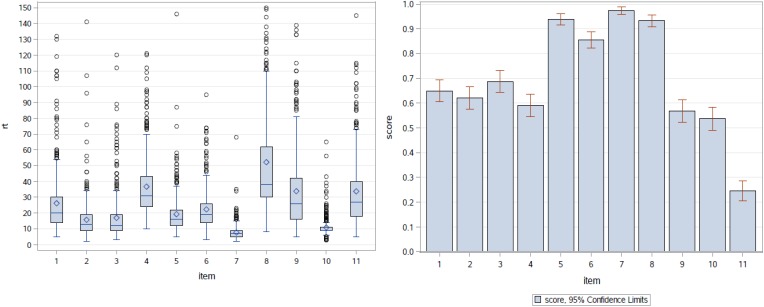
Boxplots of RTs by item (left) and proportion correct by item (right).

Regarding the item-level data, Figure 1 (left panel) shows the boxplots of RTs by item (with 23 observations with RTs greater than 150 s excluded from the plot to make the RT patterns clearer to see). It is clear that the RT distributions varied across items in terms of both central location and dispersion, although the majority of the RTs were below 50 s for all items except Item 8 (this item took more time relative to other items). These RTs were generally short, as compared to those in the traditional educational assessments discussed in the RT literature. As depicted in Figure 1 (right panel), the items were easy for the test takers. All of the items, except the last one, had a proportion correct greater than 0.5 (four were above 0.85). Items 7 and 8 present a clear contrast concerning time-consumption and difficulty—both items were very easy; but for the majority of the test takers, Item 7 could be answered in 10 s, while Item 8 took about 30 to 62 s. As shown in Table 1, these two items are very different in terms of item type: Item 7 is a single-selection MC item, while Item 8 is a simulation item.

**FIGURE 2:**
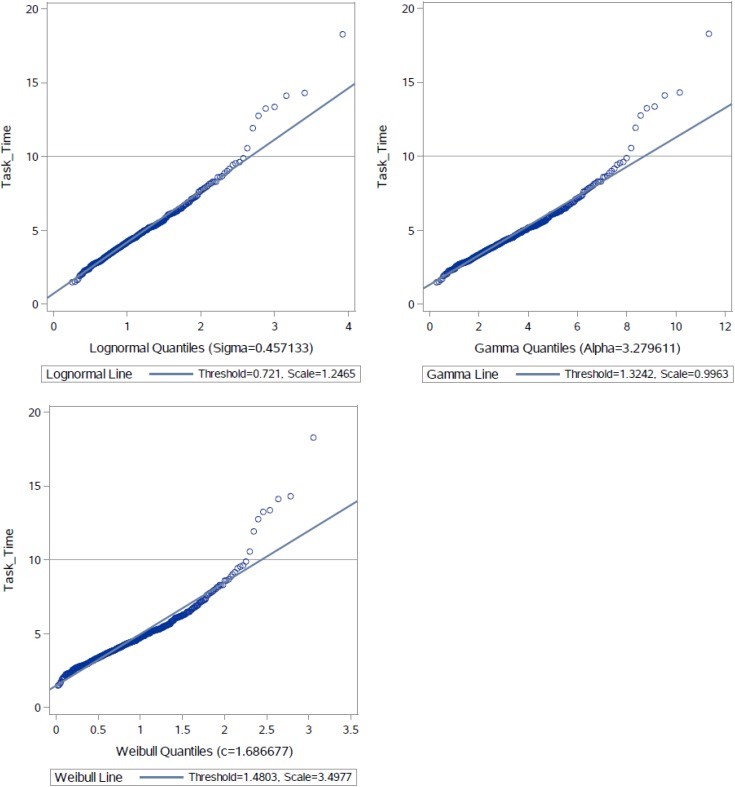
QQ plots for task times against three theoretical models.

Regarding the distribution of the timing data, the empirical timing distributions were compared to three theoretical models—lognormal model, gamma model, and Weibull model. Figure 2 presents three QQ plots that compared the empirical distribution function of the task times with the best fitting distribution of the three models. Among the three QQ plots, the lognormal model approximated the task times very well and outperformed the other two models—all of the points lay on the reference line except for 8 outliers (<2%) at the right tail. Results of the Kolmogorov-Smirnov test also suggest that the lognormal model supported the observed task times. The Kolmogorov-Smirnov test statistics for the best fitting lognormal model and gamma model were equal to 0.04 (*p*-value = 0.13) and 0.06 (*p*-value < 0.001). Similarly, the lognormal model generally supported the RTs per item, although different central locations and dispersion levels should be considered for different items. Overall, results from this analysis indicate that simple statistical models, such as lognormal regression, are appropriate for modeling the task times (Analysis 2) and for modeling the item RTs (Analysis 3) in this task.

## Analysis 2: How Did Task Times Relate to Performance and Other Information Available for the Test Takers?

As noted in the Data section, additional data were available for the test takers. Because the focus of this study was on the test takers’ timing data, variables derived from the additional information, including task score, were used as person covariates (i.e., predictors) in this analysis to investigate their relationship with the task times. The research question was to what extent the variations in the task times can be explained by these person covariates.

### Methods

To examine the effects of the person covariates on the task times, normal linear regression was employed to fit the log-transformed task times with different sets of predictors^2^. There were 27 possible covariates for the test takers:

• Two scores, one on the task and the other on the MC test. The correlation between these two scores was equal to 0.43 (*p*-value < 0.0001).
• Twelve demographic variables, including age, gender, ethnicity, high school type, experience in science, career plan after college, and home environment (related to science learning). All but “age” were treated as nominal variables.
• Three variables from the motivation questions—did you find the task engaging? Did you find the task interesting? Did you learn something new from the task? All were treated as nominal variables, each with three categories^3^.
• Ten personality variables from the personality survey. All were treated as nominal variables, each with five categories^4^.

Three models were considered. There was a base model that only included an intercept and no predictor. Model 1 included an intercept and eight predictors that were chosen subjectively from the 27 possible covariates. The eight predictors were the task score, age, gender, ethnicity, career plan after college, and the three motivation variables. Compared to the rest of the person covariates, these eight predictors are more commonly available in different large-scale educational assessments, so their effects on the task times were of interest and assessed in Model 1. The second model concerns a stepwise regression (Draper and Smith, 1998, ch. 15) that identified useful predictors from all 27 possible person covariates. The predictors were added one by one to the model only if the *F* statistic for a predictor was significant at the 0.05 level, which is recommended by Draper and Smith (1998), p. 342) for stepwise linear regression. The same criterion was used for removal of predictors. The final model is referred to as Model 2. The residual root mean squared error (RMSE), the estimated coefficient of determinationR^2^, and the estimated adjusted R^2^ were reported for each model. The RMSE represents the variability of the log-transformed task times once all useful predictors are included. Adjusted R^2^ was considered, because it combines information about model fit with number of parameters. Other measures, such as information criteria (Akaike, 1974; Schwarz, 1978), might be employed for the same purpose.

### Results

Table 2 summaries the model-fitting results. The stepwise regression approach selected 4 predictors out of 27 and outperformed Model 1, in which the 8 predictors were chosen subjectively. The final 4 predictors in Model 2 and the estimated effects on the (log-transformed) task times are as follows:

**Table 2:** Model-fitting results for task times.

Model	Number of predictors	Model degrees of freedom	RMSE	R^2^	Adjusted R^2^
Base	0	0	0.38	0.00	0.00
Model 1	8	14	0.37	0.05	0.02
Model 2	4	11	0.36	0.08	0.06

*The model degrees of freedom refers to the number of coefficients associated with the predictors and does not include the intercept.*

• Career plan after college? Test takers who worked full time or attended graduate school tended to have shorter task times than those with other plans.
• Personality variable—disorganized/careless? Test takers who strongly agreed that they were disorganized/careless spent less time than those who did not agree strongly.
• How many books at home? Test takers with enough books to fill one shelf, 11–25, tended to spend longer task times than did those with fewer or a lot more books.
• Did you find the task interesting? Test takers who chose 2 spent slightly less time than those who chose 1 (agreed most) or 3 (agreed least) did.

Although interesting, Model 2 explained only about 6% of the variability in the log-transformed task times and did not substantially reduce the RMSE relative to the base model. It was therefore concluded that none of the person covariates available in the dataset had clear effects on the test takers’ time on task, and further details about the parameter estimates in Model 2 are omitted.

## Analysis 3: How Did Item Rts and Responses Relate to Each Other?

There are many ways to examine the relationship between the observed RTs and item responses. If one assumes that the task may measure two latent traits per test taker, ability and speed, then a possible approach is the hierarchical framework for joint modeling item responses and RTs (van der Linden, 2007). This framework assumes that each test taker operates at fixed levels of speed and ability in a test. It tends to be adequate for tests with generous time limits (van der Linden, 2007, p. 292) or without any time limits—that is, the task under study.

### Methods

The hierarchical framework assumes that the task measures two latent traits for each test taker *j*, one for ability θ_j_ and the other for speed τ_j_ which may be correlated among a group of test takers of size *J* = 445. It also assumes that each item *i*, 1 ≤ *i* ≤ *I* = 11, can be characterized by such parameters as difficulty *b_i_*, time-intensity β_i_, time-discrimination α_i_, and so on, some of which may be correlated among items in a test. Let Y _ji_ and T_ji_ be test taker *j*’s response and RT on item *i*, respectively. The hierarchical framework assumes that, conditioning on the parameters for test takers and for items, item responses Y _ji_ and RTs T_ji_ on the task items are independent and can be modeled separately at Level 1 of the framework by an IRT model (for item responses Y _ji_) and a timing model (for RTs T_ji_). At Level-2 of the framework, the correlation between person parameters (i.e., ability θ_j_ and speed τ_j_) across test takers and the correlations between item parameters across items are captured in the multidimensional prior distributions and can be estimated from the data.

Due to the small sample size and short test length, the Rasch model was employed to model item responses Y _ji_, with the conditional probability equal to

$$p(Y_{\mathrm{ji}}|\theta_{j},\;b_{i})=\frac{1}{1+\exp[-(\theta_{\text{j}}-b_{i})]}.$$

According to the results in Analysis 1, the item RTs supported a lognormal model reasonably well but tended to have different central locations and levels of dispersion in the distributions for different items. Thus, a lognormal regression model with two item parameters, one for time-intensity β_i_ (to describe possible differences in the central location) and the other for time-discrimination α_i_ (to describe possible differences in the dispersion), was chosen to model the RTs. More specifically, the regression of the logarithm of T_ji_ on test taker *j*’s speed τ_j_ and item *i*’s time-intensity β_i_ may be expressed as

$$\log(T_{\mathrm{ji}})=\beta_{\text{i}}-\tau_{\text{j}}+\varepsilon_{\mathrm{ji}}$$

where the random error ε_ji_ ∼ *N*$\left(0,\alpha_{\text{i}}^{-2}\right)$. Parameter τ_j_ indicates the speed of test taker *j*, larger τ_j_ for faster respondents. Parameter β_i_ represents the time-intensity of item *i*: the larger the β_i_, the more time item *i* requires for the test takers to respond. Parameter α_i_ represents the discriminating power of item *i* in RTs, and larger α_i_ corresponds to less variable T_ji_ across test takers. The probability density function (PDF) of T_ji_ is equal to

$$f(t|\tau_{\text{j}},\beta_{\text{i}},\alpha_{\text{i}})=\frac{\alpha_{\text{i}}}{t\sqrt{2\pi }}\exp\left\{-\frac{\alpha_{\text{i}}^{2}}{2}{\left[\log(t)-\left(\beta_{\text{i}}-\tau_{\text{j}}\right)\right]}^{2}\right\}.$$

Level-2 of the framework involves joint models of the person parameters and of the item parameters. The joint distribution of the test taker’s ability θ_j_ and speed τ_j_, 1 ≤ *j* ≤ *J*, was assumed to follow a bivariate normal distribution,

$$\left(\begin{array}{c} \theta_{\text{j}}\\ \tau_{\text{j}} \end{array}\right)∼N_{2}\left(\mu_{p},\;\Sigma_{p}\right)$$

with the mean vector *μ_p_* = (0, 0)′ and covariance matrix

$$\Sigma_{p}=\left(\begin{array}{cc} \sigma_{\theta }^{2} & \sigma_{\theta \tau }\\ \sigma_{\theta \tau } & \sigma_{\tau }^{2} \end{array}\right).$$

Let ρ_θ*t*_ = σ_θ*t*_/(σ_θ_σ_t_) be the correlation between ability θ_j_ and speed τ_j_ across *j*. Similarly, for item parameters, a bivariate normal distribution was assumed for item difficulty *b_i_* and time-intensity β_i_, 1 ≤ *i* ≤ *I*,

$$\left(\begin{array}{c} b_{i}\\ \beta_{\text{i}} \end{array}\right)∼N_{2}(\mu_{I},\;\Sigma_{I})$$

with the mean vector μ_*I*_ = (μ_b_, μ_β_)′ and covariance matrix

$$\Sigma_{I}=\left(\begin{array}{cc} \sigma_{\text{b}}^{2} & \sigma_{\text{b}\beta }\\ \sigma_{\text{b}\beta } & \sigma_{\beta }^{2} \end{array}\right).$$

Let ρ_bβ_ = σ_bβ_/(σ_b_σ_β_) be the correlation between difficulty *b_i_* and time-intensity β_i_ across *i*. The item parameter moments were constrained from the general case, which includes time-discrimination α_i_ in the item parameter vector. In this study, time-discrimination α_i_ was estimated separately. The α_i_ was assumed to be independent of *b_i_* and β_i_ for two reasons. First, previous studies (e.g., Bolt and Lall, 2003; Fox et al., 2014) indicate that the correlations between the time-discrimination α_i_ and the other item parameters (*b_i_* and β_i_) provide negligible information about the item quality or person latent traits, especially the relationship between speed and accuracy among test takers. Thus, by following the convention of jointly estimating an RT model and an IRT model, the covariances related to time-discrimination α_i_ were ignored. Second, forcedly estimating the covariances related to time-discrimination α_i_ might cause an over-fitting issue with complex hierarchical modeling, which might yield untrustworthy person parameter estimates. Thus, the mentioned constraints were applied.

A software program that implements a Bayesian MCMC approach with Just Another Gibbs Sampler (JAGS; Plummer, 2015) was employed to estimate the model parameters (Man et al., 2019). The prior distributions for estimating the mean vector and the covariance structure of the item difficulty and time-intensity were specified as follows:

μb∼N(0, 2), μβ∼N(4.5, 2), ΣI∼IW (II0, νI0),and αi∼InvGamma (1, 1),

where *IW* denotes the inverse-Wishart distribution, *InvGamma* denotes the inverse-gamma distribution, *I*_*I*0_ is a 2 × 2 identity matrix, and *ν*_*I*0_ indicates the degree of freedom, which in this case is 1. Likewise, the prior distribution for estimating the covariance structure of the person parameters is defined as Σ_*p*_ ∼*IW* (*I*_*I*0_, *ν*_*I*0_), the same distribution as Σ_*I*_ given above. Model parameters were estimated by the posterior mean, or the expected *a posteriori* (EAP) estimate, through the algorithm.

The *R2jags* package (Su and Yajima, 2015) was utilized to run JAGS in R (R Core Team, 2016). The potential scale reduction (PSR) factor was used for evaluating the model parameter convergence (Gelman et al., 2003).

### Results

**FIGURE 3:**
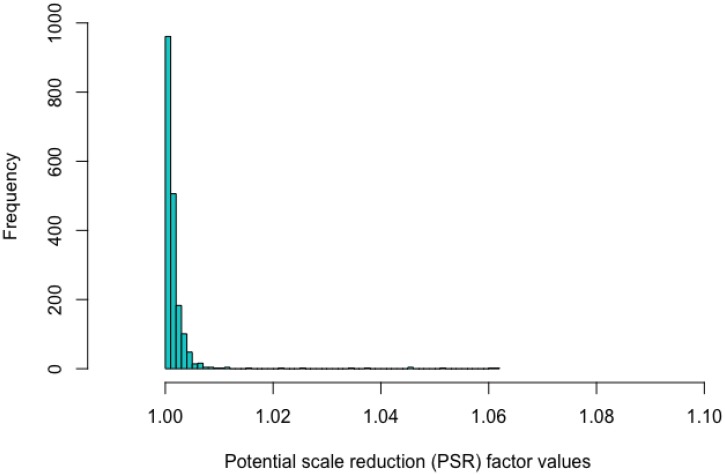
Histogram of the values of the potential scale reduction factor based on the fitted joint model.

For parameter estimation with this dataset, the MCMC approach involved two chains, each with thinning of 5 using 15,000 total iterations with a 5,000 burn-in. In this study, a PSR value of a parameter estimate lower than 1.1 indicates satisfying convergence (Gelman and Rubin, 1992a,b). Figure 3 shows that the estimation of all of the parameters converged, as all the PSR values were lower than 1.1. The current choice of hyperpriors *N* (0, 2) and *N* (4.5, 2) for μ_b_ and μ_β_ seemed suitable for the dataset with the use of the Rasch model and the two-parameter lognormal RT model as the two chains reached their convergence. Also, the current setting of priors follows the convention of fitting IRT and RT models with Bayesian estimation (e.g., van der Linden et al., 2010; Natesan et al., 2016; Luo and Jiao, 2018). However, whether such hyperpriors generally work for jointly modeling RTs and responses in the hierarchical framework needs to be addressed by additional sensitivity analysis.

**FIGURE 4:**
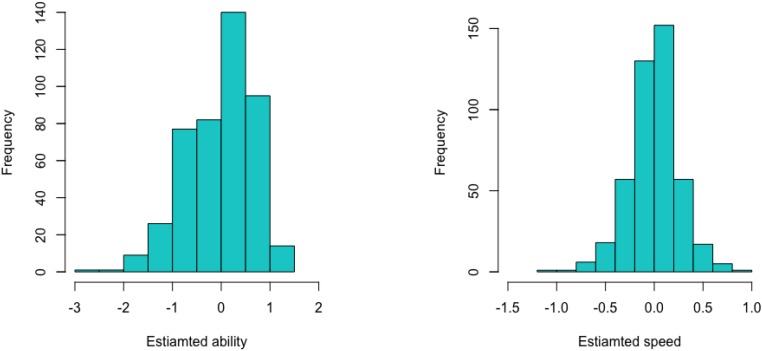
Histograms of the EAP estimates of the ability parameters (left) and of the speed parameters (right) for the test takers in the dataset.

The histogram of the EAP estimates of the ability parameters (Figure 4, left panel) was skewed to the left, while the histogram of the EAP estimates of the speed parameters (Figure 4, right panel) was roughly symmetric. Both histograms had mean equal to 0 due to the imposed constraints for identifiability of the model parameters, but the EAP estimates of the ability parameters were much more variable than were the EAP estimates of the speed parameters.

On the other hand, there was a tiny, but statistically significant, positive correlation between the ability and speed parameters among the test takers. Based on the estimated Level-2 model parameters in Table 3, the estimated correlation ${\hat{\rho }}_{\theta t}={\hat{\sigma }}_{\theta t}/\left({\hat{\sigma }}_{\theta }{\hat{\sigma }}_{\text{t}}\right)$ = 0.04/(0.85 · 0.08)^1/2^ = 0.17, with a 95% credible interval (0.034, 0.302). A positive correlation between the ability parameter and the speed parameter for a test-taker sample implies that more proficient test takers tended to work faster on the task. This level of correlation is very weak compared to many reported studies based on the same hierarchical framework. For instance, Klein Entink et al. (2009) reported an estimated correlation of −0.76 for a low-stakes assessment and an estimated correlation of 0.3 for a personality questionnaire; Wang et al. (2013) found an estimated correlation of 0.71 for a high-stakes adaptive test; Zu et al. (2016) showed estimated correlations of 0.59 for a high-stakes Listening test and of 0.86 for a high-stakes quantitative reasoning test. The authors noted that the correlation between ability and speed probably depends on the test context and content, type of test, type of item, and the test conditions. There are many possible reasons for the finding of a weak positive correlation observed in this dataset, such as different item types among the 11 items (especially simulation items vs. others), no time limit on the task, and not a challenging task to the test takers so that spending more or less time did not affect the accuracy of their responses substantially.

**Table 3:** Estimates of item parameters and level-2 model parameters.

	RT						Rasch	
	Time-intensity			Time-discrimination			Difficulty	
	EAP	SE		EAP	SE		EAP	SE
*β*_1_	3.06	0.02	*α*_1_	4.02	0.30	b_1_	−0.72	0.12
*β*_2_	2.60	0.03	*α*_2_	5.12	0.37	b_2_	−0.58	0.11
*β*_3_	2.59	0.03	*α*_3_	3.68	0.27	b_3_	−0.93	0.12
*β*_4_	3.49	0.02	*α*_4_	7.58	0.57	b_4_	−0.43	0.12
*β*_5_	2.81	0.02	*α*_5_	6.28	0.46	b_5_	−3.07	0.22
*β*_6_	2.96	0.02	*α*_6_	5.59	0.40	b_6_	−2.05	0.16
*β*_7_	1.94	0.02	*α*_7_	7.45	0.57	b_7_	−3.90	0.30
*β*_8_	3.76	0.02	*α*_8_	3.80	0.27	b_8_	−2.94	0.22
*β*_9_	3.27	0.03	*α*_9_	2.65	0.19	b_9_	−0.32	0.11
*β*_10_	2.31	0.02	*α*_10_	10.03	0.83	b_10_	−0.18	0.11
*β*_11_	3.31	0.03	*α*_11_	3.16	0.23	b_11_	1.30	0.13
**Covariance matrix of item parameters**								
$\sigma_{\text{b}}^{2}$	2.77						
*σ*_bβ_	0.24						
$\sigma_{\beta }^{2}$	0.43						
**Covariance matrix of person parameters**								
$\sigma_{\theta }^{2}$	0.85						
*σ_θτ_*	0.04						
$\sigma_{t}^{2}$	0.08						

*For each item parameter, the EAP and SE are the posterior mean and posterior standard deviation, respectively.*

**FIGURE 5:**
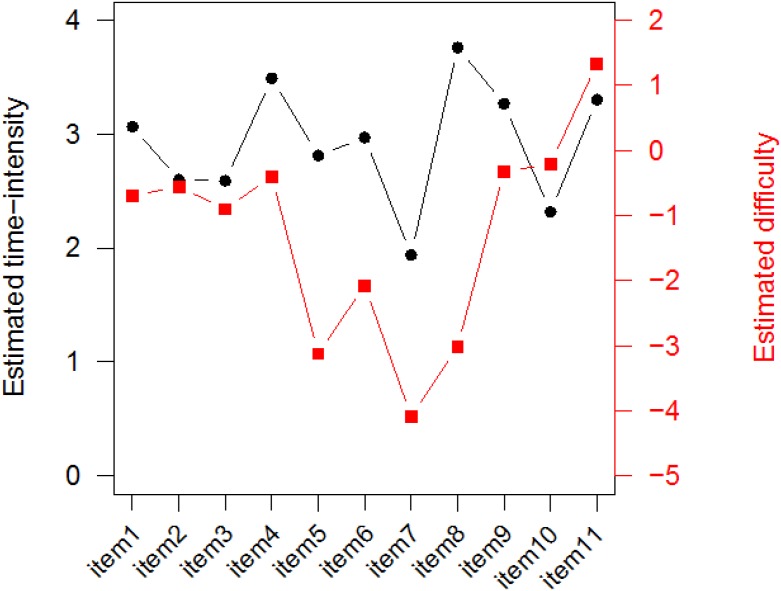
EAP estimates of item difficulty and time-intensity by item.

Based on the estimated Level-2 model parameters in Table 3, the estimated correlation between the items’ difficulty and time-intensity, ${\hat{\rho }}_{\text{b}\beta }={\hat{\sigma }}_{\text{b}\beta }/\left({\hat{\sigma }}_{\text{b}}{\hat{\sigma }}_{\beta }\right)$ = 0.24/(2.77 · 0.43)^1/2^ = 0.22 with a 95% credible interval (−0.376, 0.710). Thus, there was no clear relationship between the items’ difficulty and time-intensity. Item type is likely a key factor for this finding. In addition to the Level-2 model parameters, Table 3 also summarizes the estimates of all item parameters. To better associate the combinations of the estimated item difficulty and time-intensity with the 11 items, Figure 5 depicts their EAP estimates by item. For example, the least time-consuming item (Item 7, a single-selection MC item) was the easiest item in the task, but the most time-intensive item (Item 8, a simulation item) was also very easy. It is common for simulation-based tasks to include simulation items, which ask the test takers to follow specific instructions to conduct an experiment or a simulation, and such items are usually scored based on the completeness of the experiment/simulation. Relative to other item types, simulation items may not be difficult, but they are typically time-consuming. In the task under study, the most time-intensive but very easy item was indeed one such item, which asked the test takers to decide on the number and locations of seismometers to be placed around a volcano in order to collect proper data for later analyses. The simulation items in the two NAEP simulation-based tasks revealed the same pattern of time-intensive but easy (Jia and Lee, 2018).

**FIGURE 6:**
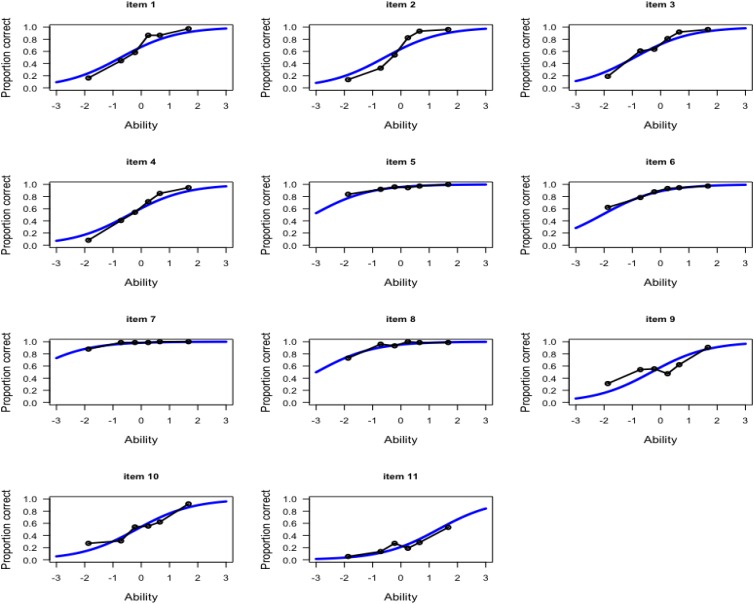
Item characteristic curve (solid blue line) with observed proportion correct (black dots line) for the 11 items.

Figure 6 presents the item characteristic curve based on the fitted Rasch model with the observed proportion correct for the 11 items. To evaluate the observed proportion correct, the test takers were classified into 6 equal-size groups based on their EAP estimates, and then the fraction of correct responses was computed per group for each item.

## Analysis 4: Did the Test Takers Show Different Timing Patterns on the Task?

The preceding section considers a parametric approach to jointly modeling item responses and RTs. The hierarchical framework in van der Linden (2007) makes assumptions that each test taker operates at fixed levels of speed and ability, and is not designed to detect different test-taking behaviors/strategies or potential latent classes. In practice, test takers may employ different strategies to allocate their time across items. Cluster analysis is a useful approach to studying different patterns of the trend and variation in RTs across items among a test-taker sample. Test takers showing similar RT patterns would be identified as a cluster. Through examination of the identified clusters, the analysis may suggest differences in strategies/behaviors across test takers and changes in strategies/behaviors across items.

### Methods

This analysis examined the RT patterns across the 11 items to look into possible trends and variations of the test takers’ response processes. Each test taker’s RT pattern spanned an 11-dimensional space, and a hierarchical cluster analysis was applied to the RT patterns of all test takers to find out how they clustered in the 11-dimensional space. After experimenting using a number of clustering methods and distance metrics, it was found that a hierarchical clustering approach with the Euclidean distance calculated from the RTs and the Ward linkage (Ward, 1963) led to the most interpretable clustering of test takers. By using the Ward linkage, a pair of clusters being chosen to be merged at each step of the hierarchical clustering process will minimally increase the total within-cluster variance. We determined the final number of big clusters based on the elbow point of the inter-cluster distances. After the clusters were identified, given a cluster, the mean of RTs was computed for each item, and the 11-dimensional mean RT vector was graphed to depict the trend and variation of the general RT pattern for the cluster. To evaluate if the clusters had different overall performance in terms of accuracy and timing or item performance, the test takers’ task times, task scores, and item responses were compared by cluster. The person covariates used in Analysis 2 were also considered for further investigation of the clusters.

### Results

**FIGURE 7:**
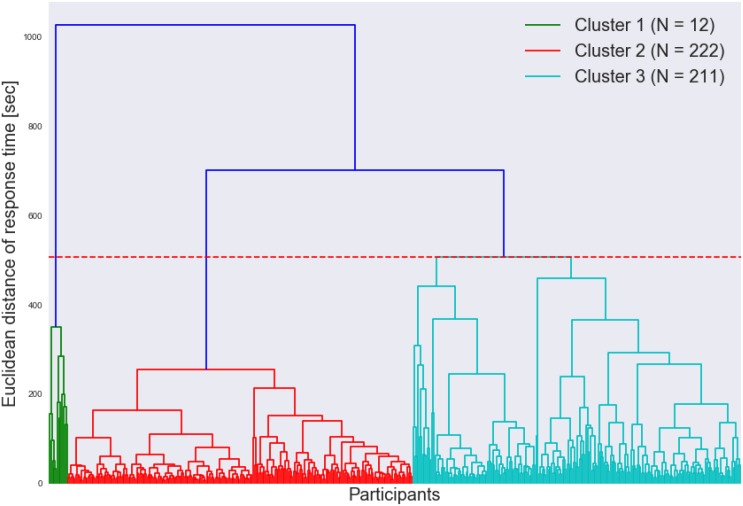
Dendrogram of the clustering of test takers based on RTs.

**FIGURE 8:**
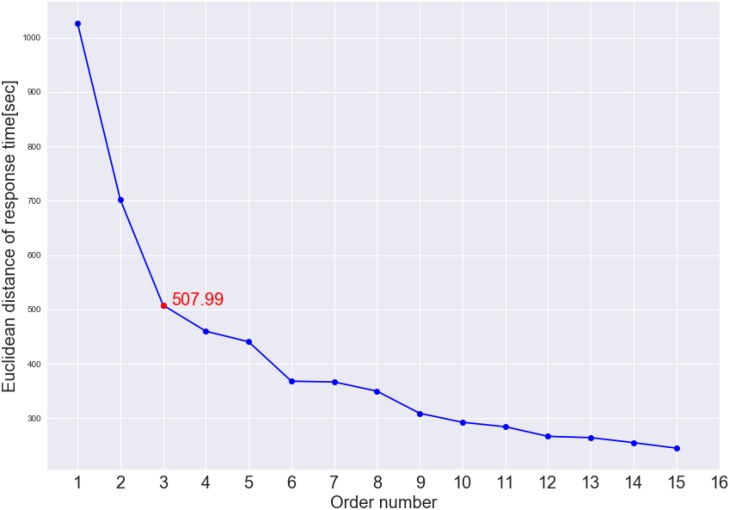
Elbow plot of the inter-cluster distance. The red dot indicates where the elbow point is located.

**FIGURE 9:**
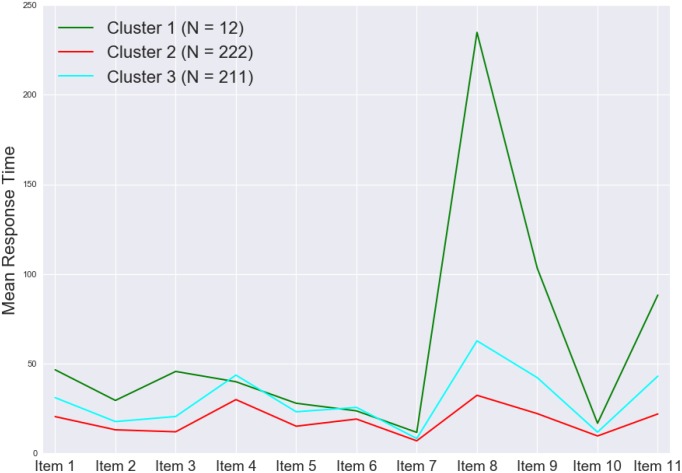
Mean RT by item for each of the three clusters.

Figure 7 shows the cluster dendrogram. Based on the elbow point of the linkage (Figure 8), three clusters were identified. For each cluster, the average time spent on each item is shown in Figure 9. One may observe that cluster 1 (with 12 test takers) corresponded to a “slow” response pattern, as those test takers spent more time on average on almost all items. Cluster 2 (with 222 test takers) corresponded to a “fast” response pattern, as the test takers spent less time on average on every item. Cluster 3 (with 211 test takers) corresponded to a “moderate” response pattern, as their average RTs lie between the average RTs of those in cluster 1 and cluster 2 on most of the items. All three clusters shared a somewhat similar timing trend on most items but deviated from the trend on specific items. The common timing trend generally follows the patterns of item time-intensity observed in the boxplots of items in Analysis 1 (Figure 1) and estimated in the hierarchical framework in Analysis 3 (Figure 5). The differences among the test takers’ RT patterns translated into different estimated speed. The existence of the three clusters with different RT patterns did not distort the RT distributions for individual items: the RT distributions of clusters 2 and 3 overlapped and did not appear as distinct peaks; cluster 1 only had 12 test takers and their RTs tended to appear as outliers in the overall RT distribution per item rather than a second mode. Thus, there was no evidence against using a lognormal distribution in modeling RTs (see section “Results” in Analysis 1), and the fact that the estimation of the model parameters converged successfully in the hierarchical modeling (Analysis 3) provided a sign of reasonable fit.

**FIGURE 10:**
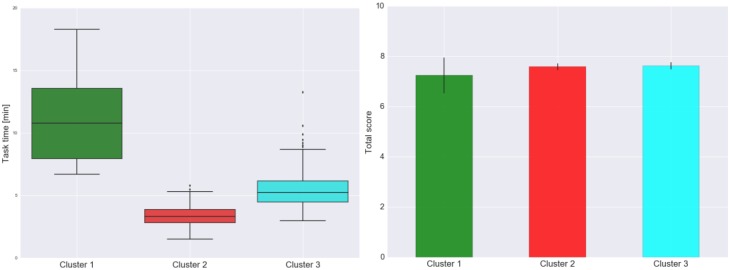
Boxplot of task times by cluster (left) and the mean task score by cluster with the associated 95% confidence limits (right).

Figure 10 shows the boxplot of task times by cluster (left panel) and the mean task score by cluster with the associated 95% confidence limits (right panel). The task-level timing differences among the three clusters agreed with the findings regarding the item-level timing patterns discussed above (Figure 9). One may find that, despite the different timing patterns, the accuracy (as reflected by the task scores) was comparable across the clusters—this result suggests that, although the test takers in different clusters might have approached the items in different ways and that resulted in differences in RTs, their performances were not much affected. This finding is consistent with the observed weak positive correlation between the test taker’s speed and ability estimated in the hierarchical framework. More importantly, results from the cluster analysis revealed variations in different clusters’ RT patterns across items, especially between cluster 1 and the rest of the test takers (Figure 9). The test takers in cluster 1 spent a lot more time to figure out what to do with Item 8, which is the simulation item that asked the participants to decide on the number of seismometers they want to use to monitor the volcano and then place them in different regions around the volcano. Besides the longer RTs on average, the test takers in cluster 1 did not do as well on Item 8 as those in clusters 2 and 3—the proportions correct for clusters 1, 2, and 3 were 0.67, 0.92, and 0.96, respectively. The 12 test takers in cluster 1 also tended to spend more time on two follow-up CR questions about the simulation (Items 9 and 11) and perform slightly worse on these items. In general, such information may be leveraged to supply valuable formative feedback to students, teachers, and assessment developers to help identify potential learning gaps or design issues. With respect to the person covariates, the only more noticeable difference among the three clusters was their gender decomposition: only one-third of cluster 1 (4 out of 12) were male, while almost two-thirds of either cluster 2 or 3 were male (which tracked the gender decomposition in the overall sample well).

## Analysis 5: Did the Test Takers Show Rapid-Guessing Behavior on the Task?

Analysis 4 employed cluster analysis to identify clusters with different timing patterns. The analysis in this section focuses on two specific test-taking behaviors, solution behavior and rapid-guessing behavior. As noted in the Introduction section, RTs have been used to differentiate rapid-guessing behavior from solution behavior. Test takers exhibiting rapid-guessing behavior on an MC item typically spend little time relative to the majority of the test takers, and their probability of answering the item correctly is likely close to the chance-level proportion correct (i.e., the expected probability of answering an item correctly by guessing). Thus, more effective approaches to identifying rapid-guessing behavior consider both item responses and RTs (e.g., Ma et al., 2011; Lee and Jia, 2014; Wang and Xu, 2015; Guo et al., 2016). There are many reasons that may lead to the presence of rapid-guessing behavior on a test: a common issue for high-stakes assessments is test speededness, whereas a common concern for low-stakes assessments is motivation. The analysis in this section is intended to assess the extent of rapid-guessing behavior in the task. Because the task was given without time limits, clear presence of rapid-guessing behavior is more likely to indicate motivation issues. If rapid guessing is negligible or not present in a dataset, then motivation is unlikely a concern.

### Methods

The non-model-based procedure in Lee and Jia (2014) was originally developed for MC tests. It was adapted by Jia and Lee (2018) to examine rapid-guessing behavior and motivation issues in the two NAEP simulation-based tasks. This procedure examines the items on a test one by one. For each item, it defines a time threshold through visual inspection of the RT distribution with the information of proportion correct evaluated at every observed RT (i.e., conditional proportion correct). For MC items, an identified time threshold for an item should classify the test takers into two groups: One group, which is assumed to exhibit solution behavior, has RTs greater than the time threshold and their proportion correct should be clearly greater than the chance level (i.e., for a 4-option single-selection MC item, the chance-level proportion correct is about 0.25). The other group, which is assumed to exhibit rapid-guessing behavior, has RTs shorter than the time threshold and conditional proportion correct close to the chance level. For items that are unlikely to be answered correctly by guessing (e.g., CR items), the chance level may be set at 0, and the rest of the procedure remains applicable (Jia and Lee, 2018).

Data with larger fractions of RTs falling below the corresponding time thresholds indicate more substantial levels of rapid guessing on the test. If no item involves the patterns of short RTs and chance-level proportion correct, or if the fraction of identified rapid guesses is negligible, then rapid guessing is considered not a concern for the test.

### Results

**FIGURE 11:**
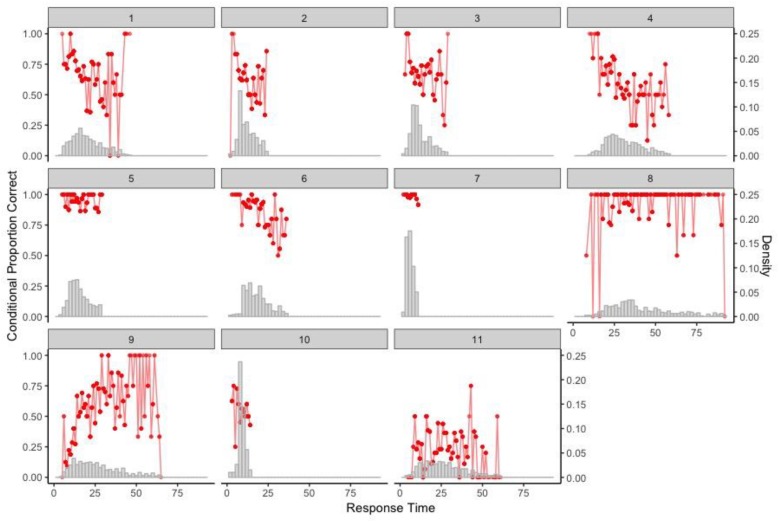
Item-level RT distribution with conditional proportion correct for the 11 items (RTs truncated at the corresponding 90th percentile).

The procedure was applied to each of the 11 items to identify possible time thresholds based on the item-level RT histograms and the associated results of conditional proportion correct. Figure 11 presents the RT distributions of all 11 items overlaid with the conditional proportion correct represented in red points. As the identification of rapid guesses focuses on shorter RTs, the RT distributions were truncated at the 90th percentile for each item. According to Table 1, the chance-level proportions correct for the MC items are as follows: 0.25 for Items 1, 2, 5, 6, and 7; 1/3 for Item 3; and 1/120 for Item 4. Items 8–11 were CR items, so their chance-level proportions correct were set at 0.

Based on Figure 11, one could, in a strict sense, identify time thresholds of 6 and 8 (seconds) for Items 9 and 11, respectively, which classified the test takers into the two behaviors—solution behavior vs. rapid-guessing behavior. However, the size of the respective resulting group for rapid-guessing behavior was almost ignorable, that is, 1 (0%) for Item 9 and 8 (<2%) for Item 11. None of the other items had an identifiable time threshold that clearly separates the two behaviors. In fact, most of the items had decent proportions correct for pretty short RTs. Thus, it was concluded that no clear rapid-guessing behavior was detected in this dataset using timing and response data, and motivation is unlikely an issue.

## Discussion

This paper presents a systematic RT study on the simulation-based task about volcano science, and investigates different timing aspects of this test-taker sample’s behavioral features at the task level and the item level. The goal is to understand the RTs in complex simulation-based tasks so as to gain insights into possible ways of leveraging RT information in designing, assembling, and scoring of simulation-based tasks. Information about the test takers’ performance on the items/task, demographics, motivation level, and personality was also considered. The task involved 11 items of various types associated with a common scenario, and was delivered without time limits. The majority of the test takers spent 6 min or less on the 11 items and performed well.

The five timing analyses revealed the following interesting findings. First, the timing data at both the task level and the item level showed good distributional properties, which made it possible to employ relatively simple statistical models that are unimodal and right-skewed, such as lognormal regression, to analyze the relationship between the timing data and other data available for the test takers. Second, the number of observations being identified as associated with rapid guessing was negligible. Thus, it was concluded that no clear rapid-guessing behavior was observed in this dataset, and motivation was not an issue for this sample-task combination. Third, the items were not time-consuming for this sample, and there was little variability in the task times for this sample. None of the available person covariate (i.e., task performance, demographics, self-reported motivation levels, and responses to personality questions) was useful in explaining the variability in the task times, so there was no notable difference in the task times among any demographic subgroups. The two major clusters identified in the cluster analysis also did not present differences in the RT patterns among the demographic subgroups. Fourth, the results of the hierarchical modeling framework indicated a weak positive correlation estimated between the test takers’ ability and speed. The three clusters identified in the cluster analysis also exhibited different RT patterns across the 11 items but comparable task scores. All three clusters shared a somewhat similar timing trend on most items but deviated from the trend on specific items. Last but not least, the hierarchical modeling framework revealed no clear association between the items’ time intensity and difficulty. The simulation item had a very different combination of difficulty and time-intensity (easy but very time-consuming) compared to the other items in the task.

There are several implications of the results concerning the design, assembly, and scoring of simulation-based tasks. First, the good distributional property of the timing variables may be attributed to the “no time limit” condition, which implies no constraint on the timing variables and that results in no missing data due to lack of time in both timing and responses. Thus, censoring, a common issue in time-to-event studies in survival analysis (see, e.g., Kalbfleisch and Prentice, 2002; Lee and Ying, 2015), is not a concern in this dataset. Imposing no time limit to a simulation-based task may allow test takers to choose their own pace in working on the items. In contrast, for tasks/tests with an overall time limit, as is the case for typical educational assessments discussed in the RT literature, the presence of time limits may lead to missing item responses and RTs, some extent of speededness, truncated times at the test level and even at the item level, or may introduce between-item dependencies among each test taker’s RTs. As a result, more sophisticated statistical models may better describe RTs and responses in time-limit tests (e.g., Ranger and Ortner, 2012; Lee and Ying, 2015; Bolsinova et al., 2017; Molenaar et al., 2018).

The finding of no clear association between the items’ time intensity and difficulty was interesting but not surprising. Among many possible factors, test type and item type may play an important role in this finding, as the simulation item had a very different combination of difficulty and time-intensity (easy but very time-consuming) compared to more traditional MC and CR items. The contrast between simulation items and more traditional MC and CR items in the time spent and difficulty was also discovered in the two NAEP simulation-based tasks studied in Jia and Lee (2018). Thus, this finding is possibly unique to simulation-based tasks, and is not necessarily generalizable to traditional educational tests with MC and/or CR items.

On the other hand, the weak positive correlation estimated between the test takers’ ability and speed in this sample suggests that task scores (or item responses) and task times (or RTs) may reveal different useful information about the test takers on the task. The cluster analysis resulted in similar conclusions. Perhaps, two scores may be reported, one about accuracy and the other about speed/efficiency, to describe a test taker’s performance on a simulation-based task. The finding of no notable difference in the task times, or in the RT patterns of the two major clusters, among any demographic subgroups indicates that fairness in terms of timing was not an issue for this sample-task combination. However, it is unclear how test takers would change their behaviors when they were told that all process data would be examined and scored. Further research is needed to evaluate such impact on person-task interactions. As already mentioned in Analysis 3, this level of correlation is unusual as compared to existing findings in the RT literature. There are many possible factors for this observation. For example, test type and item type (especially simulation items vs. others) are likely relevant. Test design and condition may be another factor—the task was delivered without time limit and was not high-stakes, so the test takers were not urged to complete accurately and quickly. Range restriction (e.g., Raju and Brand, 2003) is another possibility. The dataset under study came from a test-taker sample that seemed proficient in the task. This factor may also explain the lack of association between the test takers’ time spent on the task and the available person covariates. Further empirical studies should focus on different simulation-based tasks and/or different test-taker populations to assess the generalizability of the weak positive association observed in this study.

One potential issue with scoring the current simulation-based task is that the task length may be too short to produce reliable scores on any aspects of the task performance. The sample size was also limited in this dataset. The short task length probably results from practical constraints on the overall task time, which not only includes the time spent responding to the embedded items but also includes the time spent listening to information about the common scenario. Our study indicated that the 11 items were generally not time-consuming for this test-taker sample. Thus, it may be adequate to include a few more non-simulation items to better assess what the test takers know and can do while not making the overall task time overly excessive. Designing the simulation-based task with more items, together with a larger sample, would also open up the possibility of using more complicated statistical models to capture the more complex person-task interactions. For instance, the simulation in the task may introduce additional dependencies among the associated items, or the test takers may change their behaviors across items of different types. Extensions of the hierarchical framework (van der Linden, 2007) with more complex IRT models may better describe the additional dependencies among the associated items. Mixture models may be used to detect heterogeneous behaviors with multiple classes underlying the responses and RTs (e.g., Molenaar et al., 2018), or to detect the test takers’ shifting between solution behavior and rapid-guessing behavior with two underlying classes (e.g., Wang and Xu, 2015). Further work in this direction is worth considering.

Analysis 5 in the study concluded no notable rapid-guessing behavior or motivation issue in this dataset. Possible explanations include that this task was more engaging to this sample of test takers, the task was not too challenging to the test takers so they were willing to work on the items, and so on. Jia and Lee (2018) also found no issue with rapid-guessing behavior in the two NAEP simulation-based tasks. It is likely that simulation-based tasks are more interesting and engaging to test takers, but more research with different datasets—in terms of different tasks, different test-taker populations, different test conditions, and so on—is needed to further investigate the benefit of using simulation-based tasks in various settings. It may also be useful to retrieve more fine-grained data at the action level, including timing, processes, and others, to look further into person-task interactions (see, e.g., Ercikan and Pellegrino, 2017; Man and Harring, 2019). In any case, it will be valuable to conduct a systematic RT study similar to the one presented herein to assess different timing aspects of a test-taker sample’s behavioral features in the simulation-based task of interest. Findings from such an RT study will lead to a better understanding of the person-task interactions and therefore offer insights into possible ways to leverage RT information in designing, assembling, and scoring of the simulation-based task of interest.

## Author Contributions

Y-HL conducted analyses 1 and 2, and led the writing and revisions of the manuscript. JH supplied information about the data, conducted analysis 4, and drafted the corresponding section. KM conducted analysis 3 and drafted the corresponding section. LO conducted analysis 5 and drafted the corresponding section. All authors reviewed and revised the manuscript.

## Conflict of Interest Statement

The authors declare that the research was conducted in the absence of any commercial or financial relationships that could be construed as a potential conflict of interest.

## Footnotes

1 The original question and options were as follows: “What do you expect your main activity will be in the year after you leave college? A. Working full time; B. Attending graduate school; C. Serving in the military; D. Other.” Given the age range of the test takers, it was assumed that the test takers selected the option that best described their situation at the time they took the task, which either had occurred or had been planned.

2 Fitting normal linear regression with the log-transformed task times or fitting lognormal regression with the task times did not yield noticeable differences in the results, so only the former case was discussed.

3 The responses (1–3) to the three motivation questions were ordinal in nature. Treating each motivation question as an ordinal variable had the advantage of estimating 1 fewer parameter, but it assumed that the successive response categories were equally spaced (Long and Freese, 2006, p. 421) and had monotonic effects on the log-transformed task times. To assess the effect of this variable treatment, in addition to the models presented in Table 2, Models 1 and 2 with the three motivation variables treated as ordinal were also considered. The corresponding adjusted R^2^ were equal to 0.02 (11 degrees of freedom) and 0.05 (9 degrees of freedom), respectively. This version of Model 2 selected the same final predictors except for “Did you find the task interesting?” which did not enter the model, and this version performed slightly worse than the Model 2 in Table 2. An alternative approach was considered that replaced the three separate ordinal motivation variables with their sum score in Models 1 and 2, but this replacement also did not improve the model fit. Thus, treating the motivation questions as separate nominal variables was preferred with this dataset.

4 The test takers were asked to rate the extent to which they agreed or disagreed with the statement in each personality question from 1 (disagree strongly) to 5 (agree strongly). When treating them as ordinal variables, one could compute the Pearson correlation between the responses to a pair of questions. It was found that the Pearson correlations between any two of the 10 personality variables were below 0.4 in absolute value (i.e., weak correlation), except for two cases with correlations of −0.48 and −0.66. There was no difference in handling each of the 10 personality variables as original or nominal in Model 2 in terms of adjusted R^2^. However, replacing the 10 separate personality variables with their sum score in Model 2 led to an adjusted R^2^ equal to 0.02 (3 degrees of freedom), with only one predictor selected (How many books at home?). Thus, this version of Model 2 was not discussed further.
